# Supplementary material for: Evaluating and improving the representation of bacterial contents in long-read metagenome assemblies
Source: Genome Biol. 2024 Apr 11;25:92. doi: 10.1186/s13059-024-03234-6 (PMC11007910; doi:10.1186/s13059-024-03234-6)
Supplement: Supplementary file 7 — Additional file 7. Supplementary figures. Figure S1. Comparison of relationships between MAG yield and library size in short read libraries and HiFi libraries. Figure S2. Scatter plots showing MAG yields per gigabases (Gb) sequenced. Figure S3. Showing all bands of Fig. 5. Figure S4. K-mer spectrum plots comparing MetaBAT2 merged with rescued circles (mb-merge) and hmBin in all samples. Figure S5. K-mer spectrum plots using HRGM assemblies as the MAGs, and HiFi reads as the library. Figure S6. OTU recovery in samples, with OTU boundary set at 99%, 95% and 90%. [file 13059_2024_3234_MOESM7_ESM.pdf]

Supplementary Figures

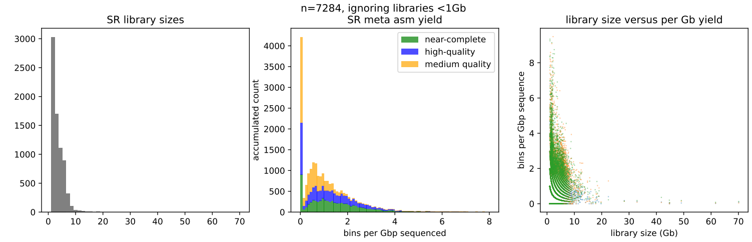

Figure S1: Almeida et al. unified assembly and MAG quality evaluations for 13,133 human gut metagenome datasets from 75 short-read-based studies. Most libraries are smaller than 10Gbp (99.2%), and per Gbp MAG recovery visually negatively correlated with library size. For an "average" library, we expect 1-2 near-complete MAGs. HiFi assemblies provide closed contigs and much lower intra-MAG heterozygosity (0.06% near-complete circular contigs have > 25% CheckM heterogeneous score, versus 23.8% for SR) at similar per Gbp yields.

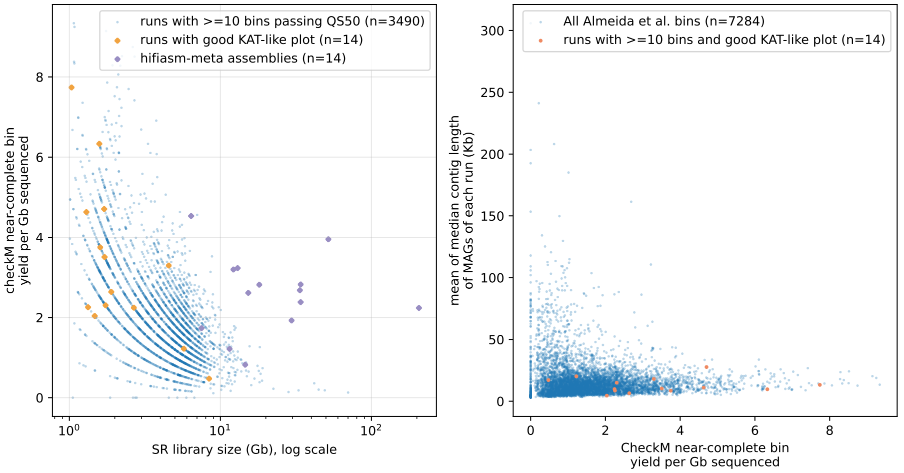

Figure S2: A few runs from short read metagenome assembly studies achieved reasonable sample representation. However, they were fragmented and larger library size did not imply a better outcome.

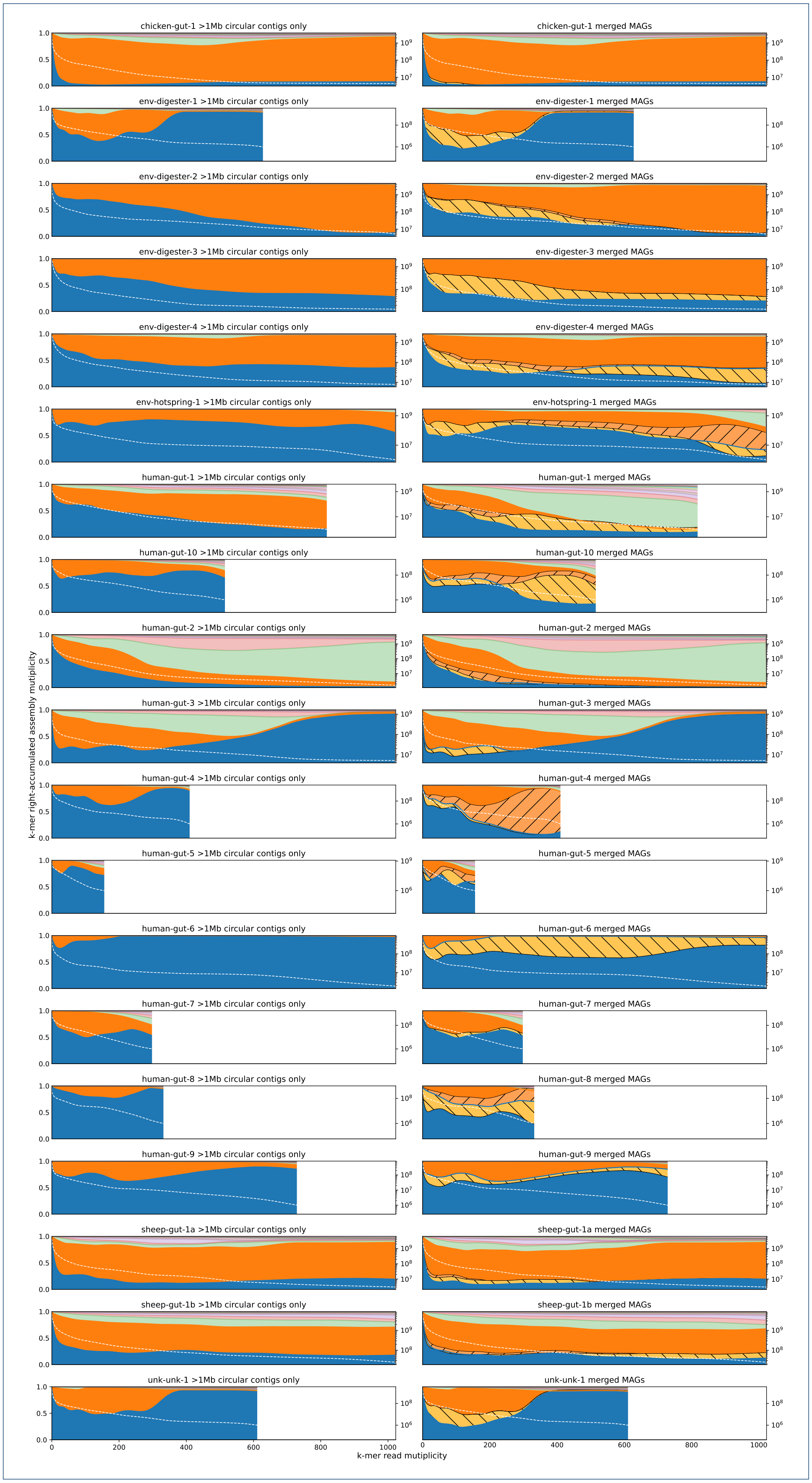
chicken-gut-1 >1Mb circular contigs only
chicken-gut-1 merged MAGs
env-digester-1 >1Mb circular contigs only
env-digester-1 merged MAGs
env-digester-2 >1Mb circular contigs only
env-digester-2 merged MAGs
env-digester-3 >1Mb circular contigs only
env-digester-3 merged MAGs
env-digester-4 >1Mb circular contigs only
env-digester-4 merged MAGs
env-hotspring-1 >1Mb circular contigs only
env-hotspring-1 merged MAGs
human-gut-1 >1Mb circular contigs only
human-gut-1 merged MAGs
human-gut-10 >1Mb circular contigs only
human-gut-10 merged MAGs
human-gut-2 >1Mb circular contigs only
human-gut-2 merged MAGs
human-gut-3 >1Mb circular contigs only
human-gut-3 merged MAGs
human-gut-4 >1Mb circular contigs only
human-gut-4 merged MAGs
human-gut-5 >1Mb circular contigs only
human-gut-5 merged MAGs
human-gut-6 >1Mb circular contigs only
human-gut-6 merged MAGs
human-gut-7 >1Mb circular contigs only
human-gut-7 merged MAGs
human-gut-8 >1Mb circular contigs only
human-gut-8 merged MAGs
human-gut-9 >1Mb circular contigs only
human-gut-9 merged MAGs
sheep-gut-1a >1Mb circular contigs only
sheep-gut-1a merged MAGs
sheep-gut-1b >1Mb circular contigs only
sheep-gut-1b merged MAGs
unk-unk-1 >1Mb circular contigs only
unk-unk-1 merged MAGs
k-mer right-accumulated assembly mutiplicity
k-mer read mutiplicity
0
200
400
600
800
1000
0.0
0.5
1.0

Figure S3: Showing all bands of Figure 5.

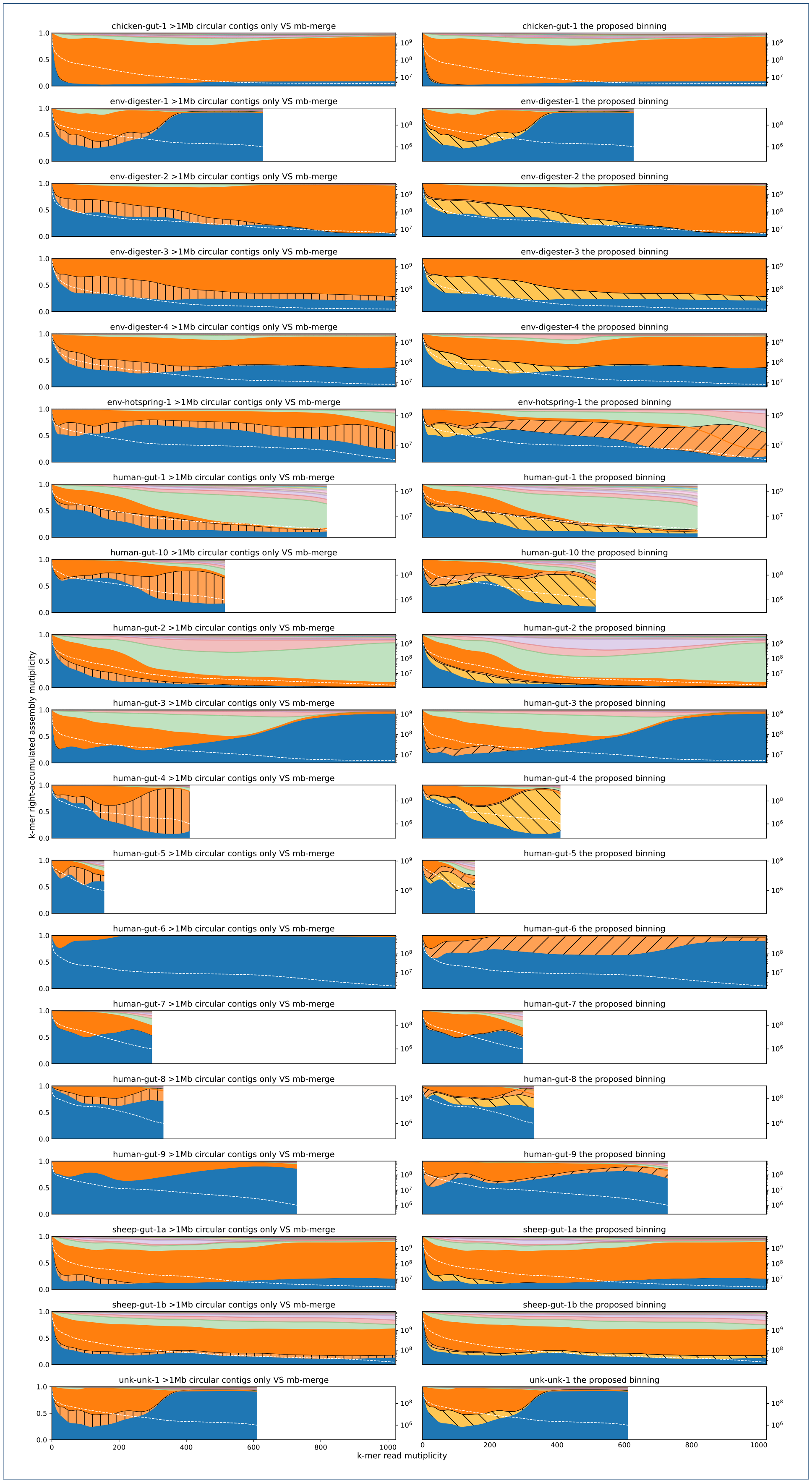
chicken-gut-1 >1Mb circular contigs only VS mb-merge
chicken-gut-1 the proposed binning
env-digester-1 >1Mb circular contigs only VS mb-merge
env-digester-1 the proposed binning
env-digester-2 >1Mb circular contigs only VS mb-merge
env-digester-2 the proposed binning
env-digester-3 >1Mb circular contigs only VS mb-merge
env-digester-3 the proposed binning
env-digester-4 >1Mb circular contigs only VS mb-merge
env-digester-4 the proposed binning
env-hotspring-1 >1Mb circular contigs only VS mb-merge
env-hotspring-1 the proposed binning
human-gut-1 >1Mb circular contigs only VS mb-merge
human-gut-1 the proposed binning
human-gut-10 >1Mb circular contigs only VS mb-merge
human-gut-10 the proposed binning
human-gut-2 >1Mb circular contigs only VS mb-merge
human-gut-2 the proposed binning
human-gut-3 >1Mb circular contigs only VS mb-merge
human-gut-3 the proposed binning
human-gut-4 >1Mb circular contigs only VS mb-merge
human-gut-4 the proposed binning
human-gut-5 >1Mb circular contigs only VS mb-merge
human-gut-5 the proposed binning
human-gut-6 >1Mb circular contigs only VS mb-merge
human-gut-6 the proposed binning
human-gut-7 >1Mb circular contigs only VS mb-merge
human-gut-7 the proposed binning
human-gut-8 >1Mb circular contigs only VS mb-merge
human-gut-8 the proposed binning
human-gut-9 >1Mb circular contigs only VS mb-merge
human-gut-9 the proposed binning
sheep-gut-1a >1Mb circular contigs only VS mb-merge
sheep-gut-1a the proposed binning
sheep-gut-1b >1Mb circular contigs only VS mb-merge
sheep-gut-1b the proposed binning
unk-unk-1 >1Mb circular contigs only VS mb-merge
unk-unk-1 the proposed binning
k-mer right-accumulated assembly mutiplicity
k-mer read mutiplicity
0
200
400
600
800
1000

Figure S4: K-mer spectrum plots of all samples, comparing MetaBAT2 merged with rescued circles (mb-merge) and hmBin. Subgraphs in the right column are identical to Figure 5's right column. In the left column, light orange regions with vertical hatches represent the contribution of SemiBin with respect to the collection of long circular contigs.

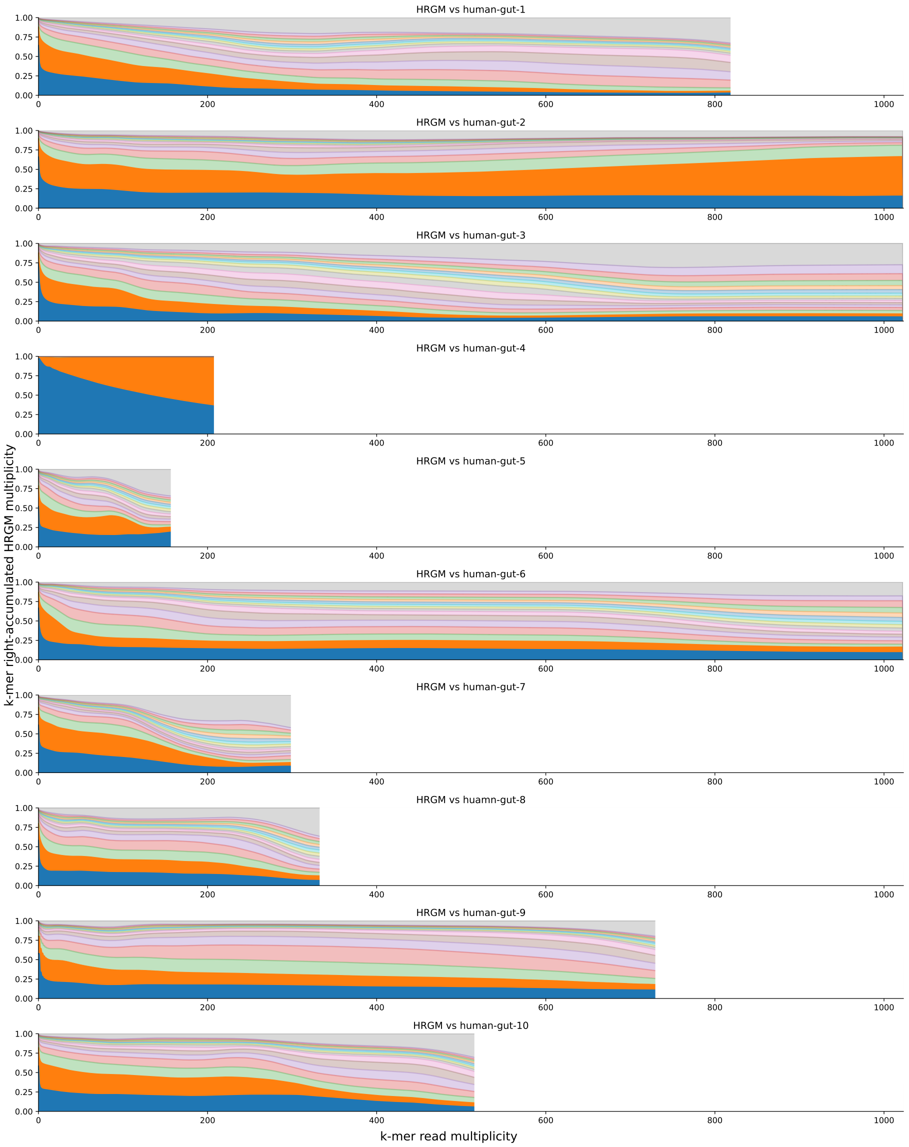

Figure S5: K-mer spectrum plots using HRGM assemblies as the MAGs, and HiFi reads as the library. It is similar to Figure 5 except that it additionally shows k-mers of higher occurrences: green areas for $N_x^{(2)}/N_x$, light red areas for $N_x^{(3)}/N_x$ and so on.

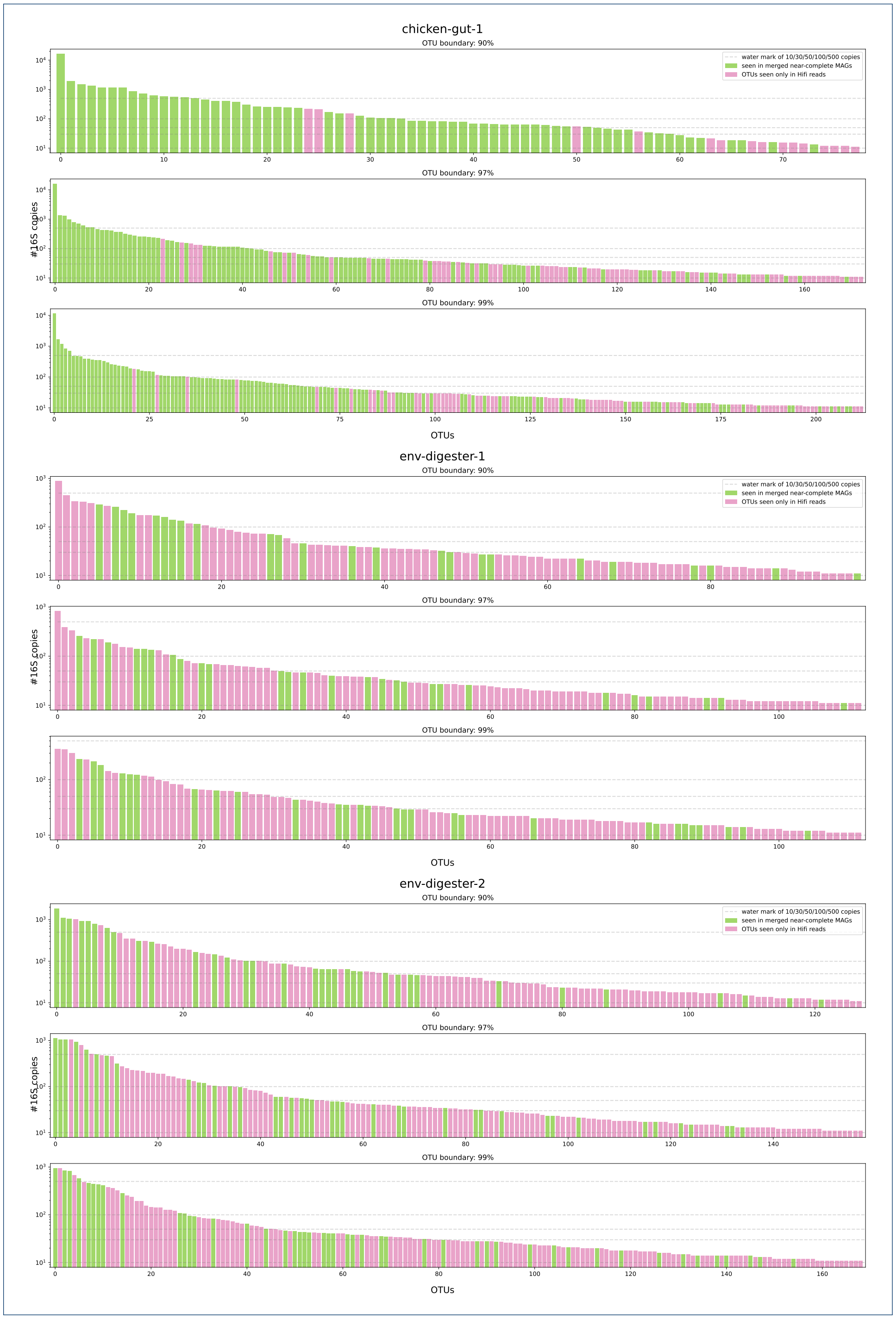

chicken-gut-1
OTU boundary: 90%
OTU boundary: 97%
OTU boundary: 99%
water mark of 10/30/50/100/500 copies
seen in merged near-complete MAGs
OTUs seen only in Hifi reads
#16S copies
OTUs
env-digester-1
env-digester-2

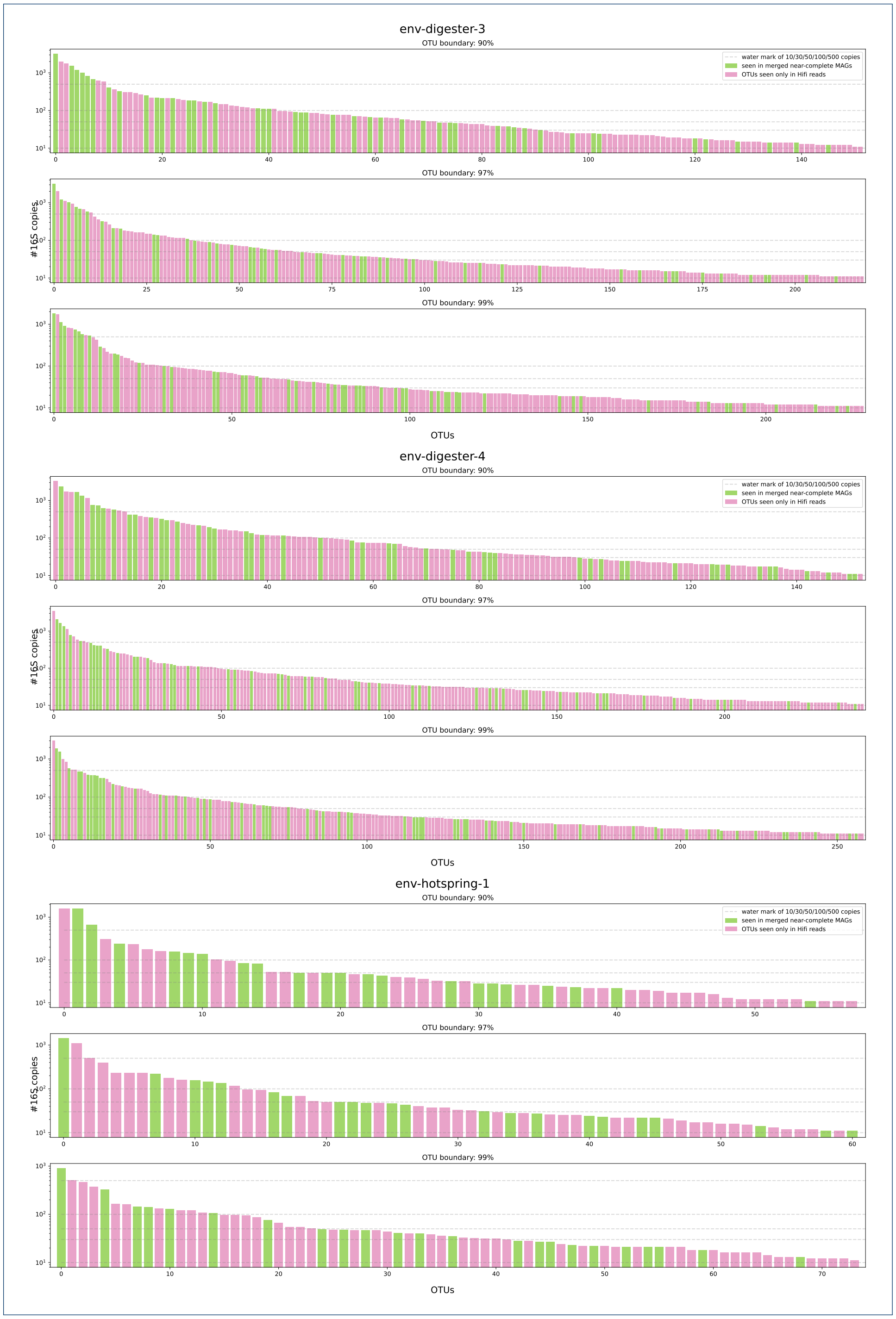
env-digester-3
OTU boundary: 90%
water mark of 10/30/50/100/500 copies
seen in merged near-complete MAGs
OTUs seen only in Hifi reads
OTU boundary: 97%
#16S copies
OTU boundary: 99%
OTUs
env-digester-4
OTU boundary: 90%
water mark of 10/30/50/100/500 copies
seen in merged near-complete MAGs
OTUs seen only in Hifi reads
OTU boundary: 97%
#16S copies
OTU boundary: 99%
OTUs
env-hotspring-1
OTU boundary: 90%
water mark of 10/30/50/100/500 copies
seen in merged near-complete MAGs
OTUs seen only in Hifi reads
OTU boundary: 97%
#16S copies
OTU boundary: 99%
OTUs

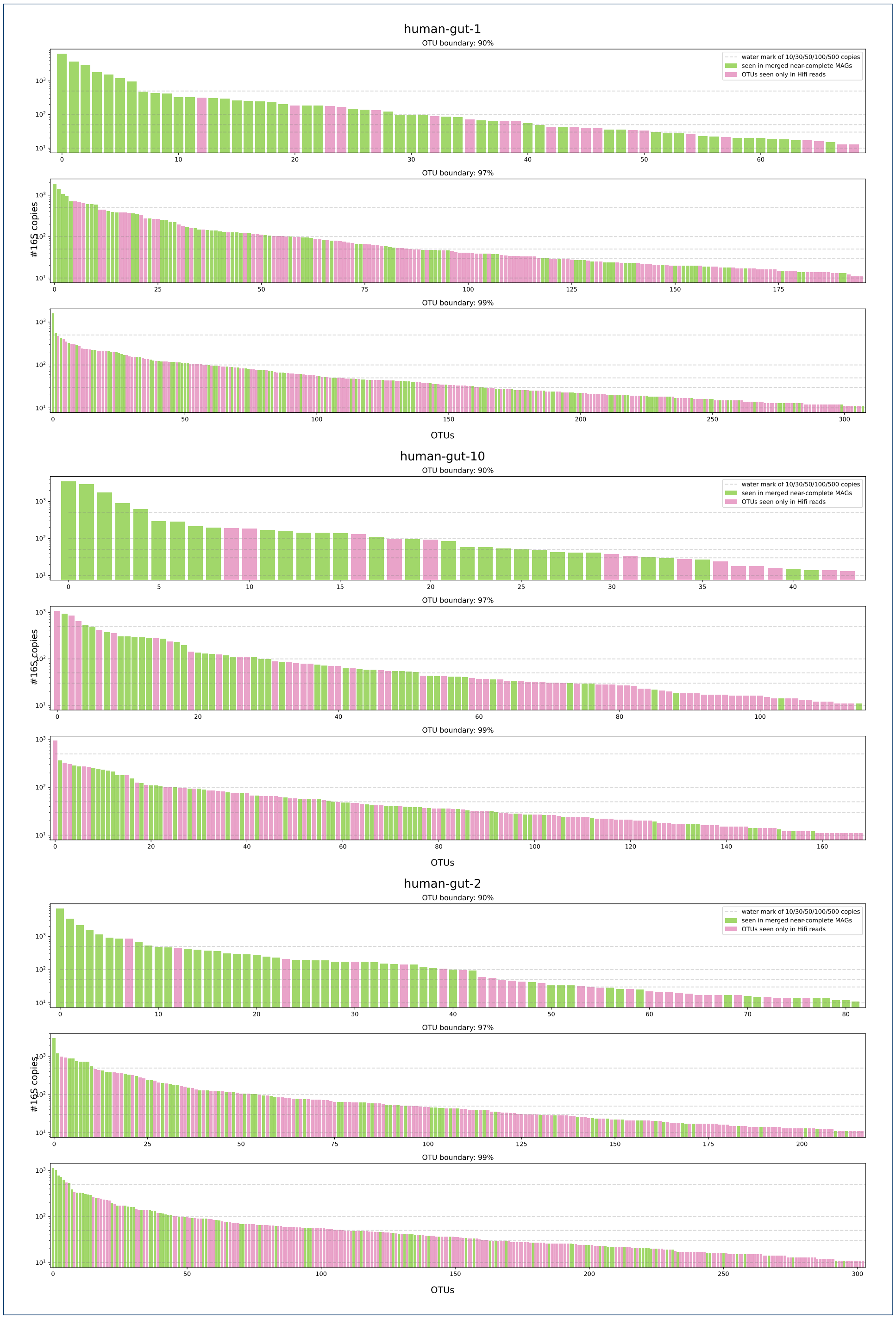
human-gut-1
OTU boundary: 90%
water mark of 10/30/50/100/500 copies
seen in merged near-complete MAGs
OTUs seen only in Hifi reads
OTU boundary: 97%
#16S copies
OTU boundary: 99%
OTUs
human-gut-10
OTU boundary: 90%
water mark of 10/30/50/100/500 copies
seen in merged near-complete MAGs
OTUs seen only in Hifi reads
OTU boundary: 97%
#16S copies
OTU boundary: 99%
OTUs
human-gut-2
OTU boundary: 90%
water mark of 10/30/50/100/500 copies
seen in merged near-complete MAGs
OTUs seen only in Hifi reads
OTU boundary: 97%
#16S copies
OTU boundary: 99%
OTUs

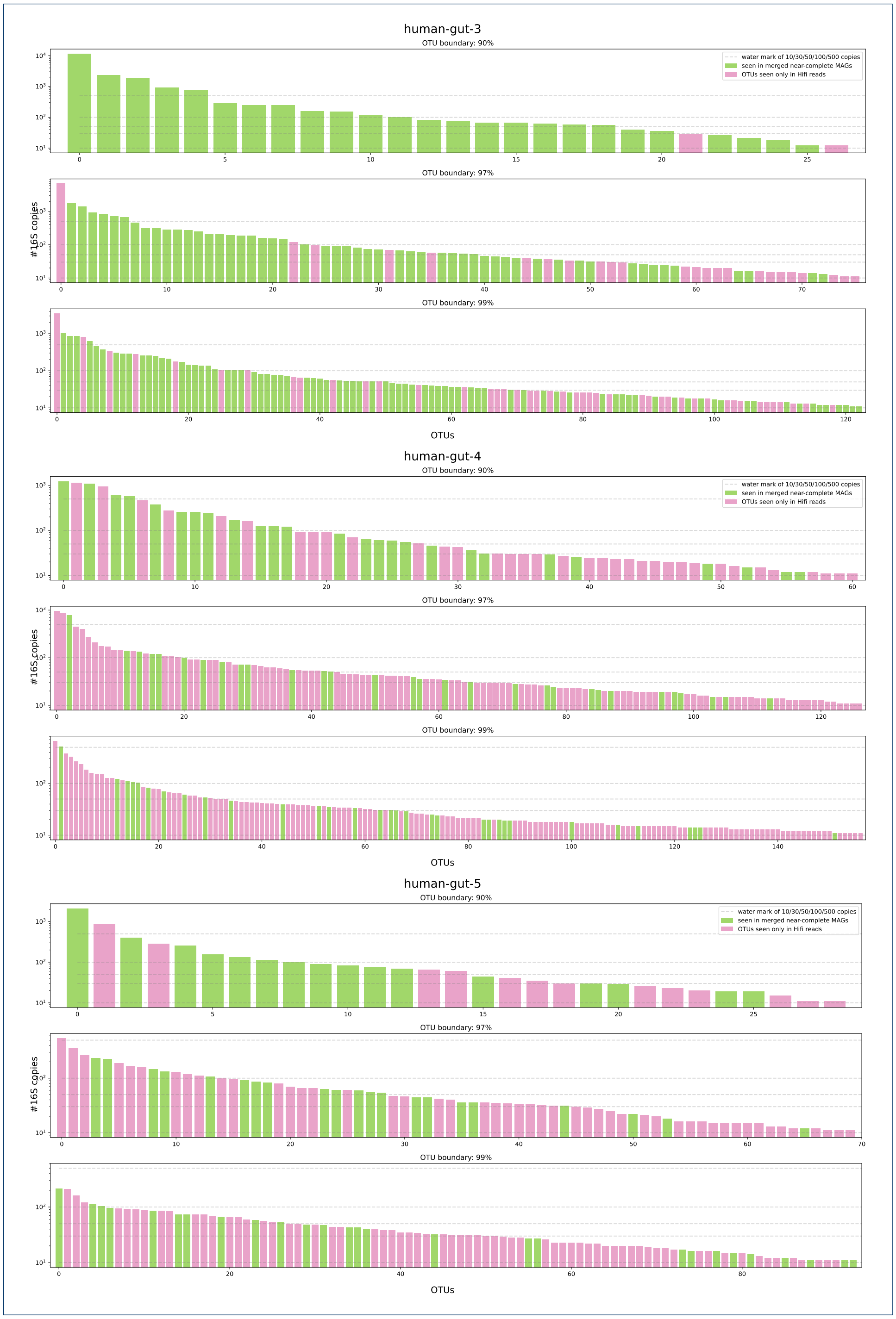
human-gut-3
OTU boundary: 90%
water mark of 10/30/50/100/500 copies
seen in merged near-complete MAGs
OTUs seen only in Hifi reads
OTU boundary: 97%
#16S copies
OTU boundary: 99%
OTUs
human-gut-4
OTU boundary: 90%
water mark of 10/30/50/100/500 copies
seen in merged near-complete MAGs
OTUs seen only in Hifi reads
OTU boundary: 97%
#16S copies
OTU boundary: 99%
OTUs
human-gut-5
OTU boundary: 90%
water mark of 10/30/50/100/500 copies
seen in merged near-complete MAGs
OTUs seen only in Hifi reads
OTU boundary: 97%
#16S copies
OTU boundary: 99%
OTUs

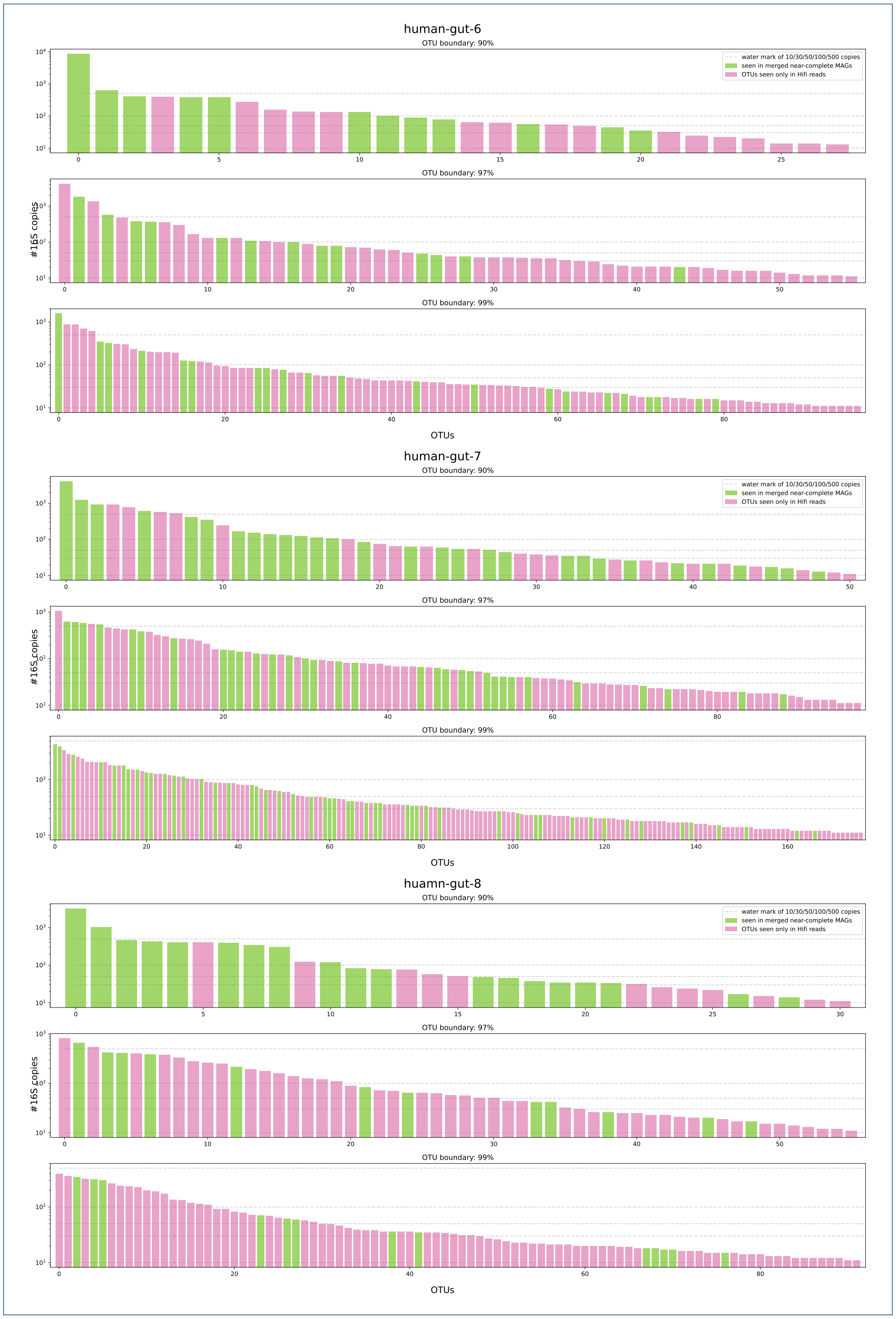
human-gut-6
OTU boundary: 90%
water mark of 10/30/50/100/500 copies
seen in merged near-complete MAGs
OTUs seen only in Hifi reads
OTU boundary: 97%
#16S copies
OTU boundary: 99%
OTUs
human-gut-7
OTU boundary: 90%
water mark of 10/30/50/100/500 copies
seen in merged near-complete MAGs
OTUs seen only in Hifi reads
OTU boundary: 97%
#16S copies
OTU boundary: 99%
OTUs
huamn-gut-8
OTU boundary: 90%
water mark of 10/30/50/100/500 copies
seen in merged near-complete MAGs
OTUs seen only in Hifi reads
OTU boundary: 97%
#16S copies
OTU boundary: 99%
OTUs

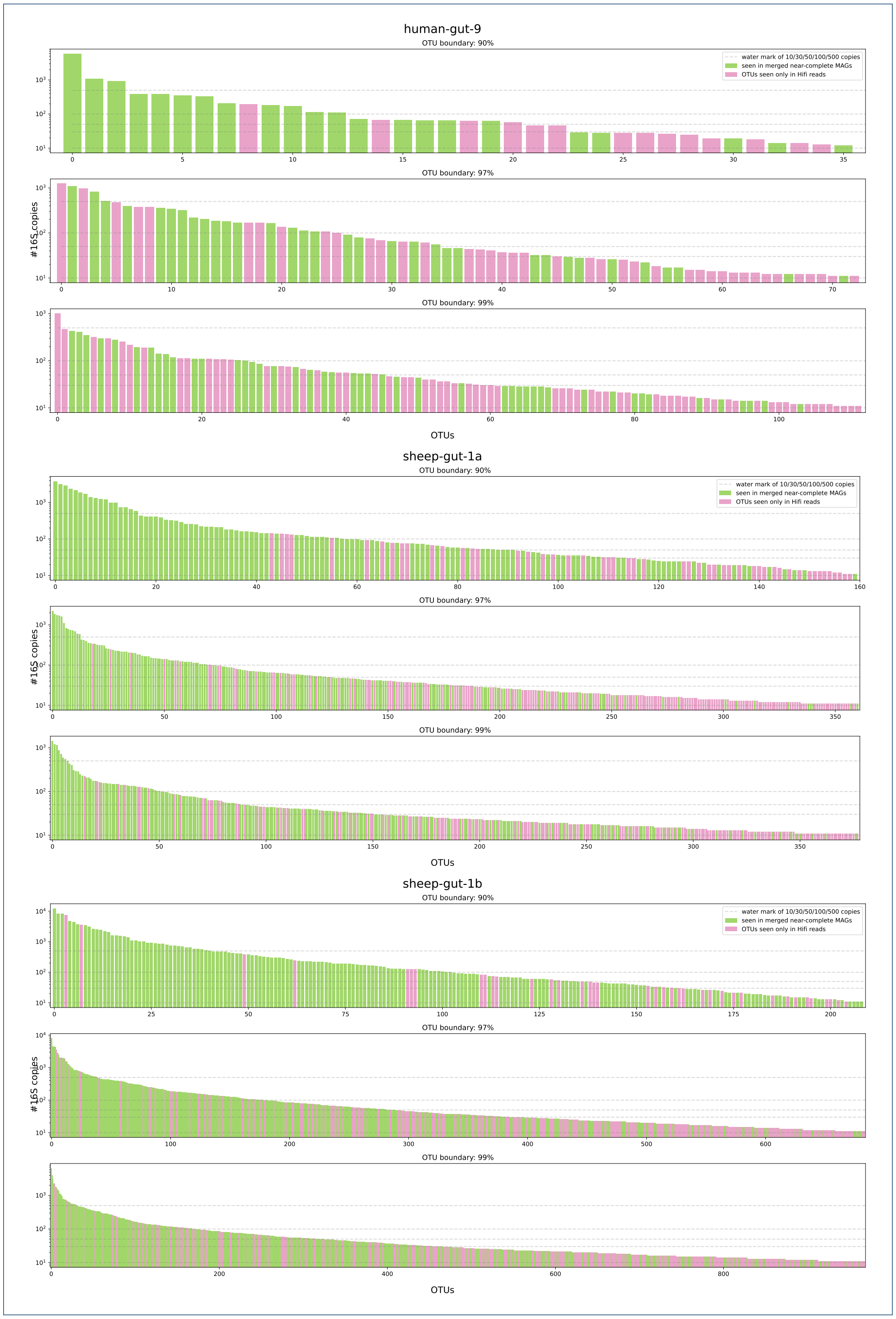
human-gut-9
OTU boundary: 90%
water mark of 10/30/50/100/500 copies
seen in merged near-complete MAGs
OTUs seen only in Hifi reads
OTU boundary: 97%
#16S copies
OTU boundary: 99%
OTUs
sheep-gut-1a
OTU boundary: 90%
water mark of 10/30/50/100/500 copies
seen in merged near-complete MAGs
OTUs seen only in Hifi reads
OTU boundary: 97%
#16S copies
OTU boundary: 99%
OTUs
sheep-gut-1b
OTU boundary: 90%
water mark of 10/30/50/100/500 copies
seen in merged near-complete MAGs
OTUs seen only in Hifi reads
OTU boundary: 97%
#16S copies
OTU boundary: 99%
OTUs

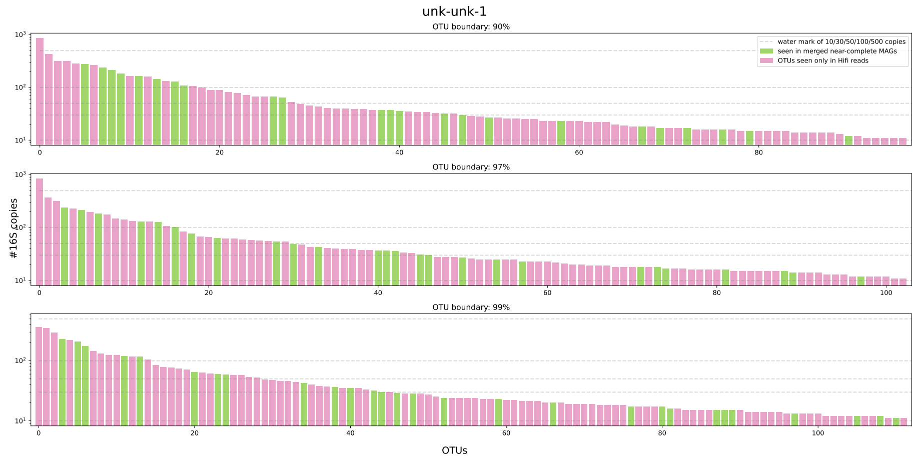

Figure S6: OTU recovery in samples, with OTU boundary set at 99%, 95% and 90%. The plots for human-gut-1 and human-gut-2 were omitted, because the each of these libraries are a pool of four different samples.
